# What can Mohs surgery do to help climate change?

**DOI:** 10.1002/ski2.26

**Published:** 2021-03-26

**Authors:** E. Butt

**Affiliations:** ^1^ Sandwell and West Birmingham Hospitals NHS Trust Birmingham UK; ^2^ School of Clinical Medicine University of Cambridge Cambridge UK

## Abstract

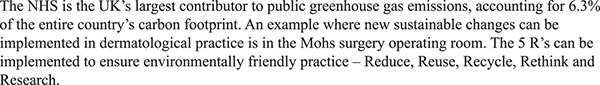

## 1 WHAT CAN MOHS SURGERY DO TO HELP CLIMATE CHANGE?

Climate change leads to increasing global temperatures, and with it comes a huge impact on dermatology. The effect on weather and ozone depletion has led to increasing rates of infectious diseases and skin cancers.^1^ The NHS is the UK's largest contributor to public greenhouse gas emissions, accounting for 6.3% of the entire country's carbon footprint. The operating theatre in particular produces up to 70% of hospital waste and emits large quantities of energy.^2^ As Mohs surgery is largely done under local anaesthetic and is a relatively short procedure, the energy costs in the operating room are better than most; however, levels of waste are still high and more can be done in the fight against climate change.

### 1.1 Reduce, reuse and recycle

As Mohs surgical packs are industry made, rather than locally, items often remain unused depending on the surgeon's personal preferences. However, once opened, they are deemed ‘exposed’ to the surgical field and become waste. At one skin centre alone, the environmental footprint of materials used in Mohs surgery estimated a yearly generation of 194 kg contaminated waste, 92 kg noncontaminated waste and 37 kg sharps waste. Extrapolating this to the 25 skin surgery centres in the UK, this equated to 644 kg of CO_2_ production from Mohs surgery alone.^3^

By collaborating with surgeons, the standard Mohs surgery sets can be individualized to a reduced list of essential items only, with additional equipment provided on request, to prevent unnecessary waste.^3^ Furthermore, it is known that most clinical waste is misallocated as offensive when it could be sent to landfill or recycled.^2^ This leads to lower levels of recycling and greater unnecessary incineration, thus contaminating the environment through the release of nitrous oxide and carcinogens. This also has huge implications for cost, as incineration incurs 86% the cost of waste due to its energy demands. Appropriate recycling and waste disposal can be fostered through simple measures. At skin centres, staff have been educated on correct waste disposal methods and the correct recycling instructions of surgical items.^3^ Also, ergonomically arranging waste bins in theatre so that nonoffensive and recycle bins are more readily available compared to offensive bins have shown a 50% reduction in medical waste volume and an increase in recycling rates.^2^

Lessons of reusing devices from abroad also have the potential to be transferred to the NHS. Disposable and single‐use devices are used in surgery to maintain sterility, but this has promoted a throw‐away culture in healthcare. Disposable devices such as the Dermatology Procedure pack, Lister forceps and Baby Metzenbaum scissors are regularly used. The US and Europe currently reprocess more than 100 types of similar single‐use devices, which have been found to be safe for use.^4^

### 1.2 Rethink and research

The era of COVID‐19 has allowed us to rethink our clinical practice. In order to reduce patient contact, the NHS has promoted a culture of digital health and virtual consultations. Compared to face‐to‐face consultations, this has led to lower rates of vehicle traffic and reduced energy usage. Virtual consultation has been used preoperatively in patients undergoing Mohs surgery. This has demonstrated higher rates of patient clinic attendance, decreased time to surgery and allowed a greater number of tumours to be operated on.^5^ Continuing the use of digital health in the future will reduce the burning of fossil fuels, improve air pollution and increase efficiency of clinical practice.

Furthermore, sustainability research in dermatology is needed to propose new ideas, analyse the effects and develop evidence‐based recommendations. For example, investigating the cost and clinical effectiveness on using a single set of instruments for both tumour removal and repair processes in Mohs surgery, can dramatically reduce waste.^6^ Future large‐scale clinical trials are needed to redesign surgical equipment and sterilization techniques, in order to reduce waste and find ways to reuse instruments. To aid research, The Centre for Sustainable Health has pioneered the use of carbon footprinting and triple bottom line analysis to evaluate environmentally friendly healthcare. Carbon footprinting measures greenhouse gas emissions, whilst triple bottom analysis measures the social, environmental and financial impact of an intervention. These values need to be routinely measured in Dermatology research to show where change is needed.

### 1.3 The 5 R’s

To be ‘environmentally friendly’ in Mohs surgery there are 5R's that can be implemented: *‘Reduce, Reuse, Recycle’* waste, as well as *‘Rethink’* practice to encourage the use of teledermatology, and *‘Research’* into new sustainable initiatives.

## CONFLICT OF INTEREST

The author declares that there is no conflict of interest
